# Factors related to changes in visual symptoms after successful photodynamic therapy in central serous chorioretinopathy

**DOI:** 10.1371/journal.pone.0284899

**Published:** 2023-04-21

**Authors:** Geun Woo Lee, Yun Young Kim, Kyung Jun Choi, Se Woong Kang

**Affiliations:** 1 Department of Ophthalmology, Daegu Catholic University School of Medicine, Daegu, Korea; 2 Department of Ophthalmology, Samsung Medical Center, Sungkyunkwan University School of Medicine, Seoul, Korea; Tsukazaki Hospital, JAPAN

## Abstract

To investigate biomarkers related to visual symptom and best corrected visual acuity (BCVA) improvement after photodynamic therapy (PDT) for central serous chorioretinopathy. This retrospective cross-sectional study involved 42 consecutive eyes, from 42 patients who underwent successful PDT, divided into two groups according to improvement in subjective visual complaints: complete (20 eyes) and incomplete recovery (22 eyes). The clinical characteristics of each group, including central foveal thickness (CFT), foveal avascular zone (FAZ) area, and degree of change in signal voiding of the choriocapillaris on optical coherence tomography angiography, were compared. Correlations between best-corrected visual acuity (BCVA) and baseline clinical features were investigated. At baseline, CFT and FAZ areas showed significant differences between the two groups (all p < 0.05). Multiple binary logistic regression analysis revealed that greater CFT predicted complete recovery from visual complaints (p = 0.002). Reduction or disappearance of signal voiding in the choriocapillaris 6 months post-PDT occurred more frequently in the complete recovery group (p < 0.05). FAZ area before PDT correlated with BCVA before and 6 months after PDT and BCVA improvement during the study period (all p < 0.05). CFT and FAZ area before PDT correlated with completeness of visual symptom recovery after PDT. Smaller FAZ area before PDT correlated with better BCVA before and after treatment.

## Introduction

Central serous chorioretinopathy (CSC) is characterized by a leakage point at the retinal pigment epithelium (RPE) level, accompanied by serous detachment of the neurosensory retina at the posterior pole [1]. It is related to choroidal vasculature abnormalities, such as congestion of the choroidal vessels and choroidal hyperpermeability [2–4]. Patients generally complain of symptoms, such as decreased visual acuity, central scotoma, metamorphopsia, and hyperopia due to subretinal fluid (SRF) at the fovea [5]. CSC occurs preferentially as an acute disease and often improves on its own. If SRF remains for 4–6 months or longer, leading to a chronic course, symptoms like decreased visual acuity and scotoma may persist permanently [6]. Photodynamic therapy (PDT) is an effective treatment for chronic CSC, and intravitreal injection of anti-vascular endothelial growth factor is ineffective [7,8]. If PDT is successful, the SRF disappears and the retinal sensitivity increases on microperimetry [9,10]. However, even after successful PDT, the degree of recovery from subjective visual symptoms (e.g., central scotoma, metamorphopsia, decreased visual acuity) may be insufficient [11,12].

Optical coherence tomography angiography (OCTA) is a noninvasive method that makes it possible to easily identify the microvasculature of the retina and choroid [13]. Several studies have used optical coherence tomography (OCT) and OCTA to study the retinal and choroidal changes that appear after PDT [11,14–17]. However, there are few studies on the clinical characteristics related to the changes in subjective visual symptoms after PDT. Despite the presence of SRF in CSC, best corrected visual acuity (BCVA) could be maintained occasionally [18]. Even though some patients with CSC complain of visual symptoms, they may have normal or near-normal visual acuity when the change in refraction caused by SRF is corrected. In light of this, it has been noted that BCVA may not be an accurate representative of the changes in visual symptoms reported by patients. The purpose of this study was to investigate the clinical characteristics that influence changes in visual symptoms and BCVA after PDT in CSC patients, using OCT and OCTA.

## Methods

### Subject selection and design

This retrospective cross-sectional study was performed at a single center and approved by the Institutional Review Board of Samsung Seoul Hospital (IRB no. 2018-07-036). The requirement for written informed consent was waived because of the retrospective design of the study, which was conducted in accordance with the tenets of the Declaration of Helsinki.

PDT was performed by a single retinal specialist. We enrolled patients who underwent PDT due to CSC, had symptoms for more than 3 months, and had been followed up without recurrence for at least 6 months. The subjects were recruited retrospectively from January 2017 to April 2019. Successful PDT was defined as complete resolution of SRF on OCT at 1 or 3 months after PDT and, at the same time, no evidence of recurrence (SRF) on OCT 6 months after PDT.

Before conducting the PDT, all subjects underwent a comprehensive ophthalmic examination, including measurements of best-corrected visual acuity (BCVA), manifest refraction, anterior segment examination using a slit lamp, dilated fundus examination, spectral-domain optical coherence tomography (SD-OCT, Spectralis HRA-OCT; Heidelberg Engineering, Heidelberg, Germany), and swept-source optical coherence tomography angiography (SS-OCTA, DRI OCT Triton; Topcon Corporation, Tokyo, Japan). Dilated fundus examination, BCVA, SD-OCT, and SS-OCTA were performed 1 and 6 months after PDT. Exclusion criteria included a history of PDT or laser photocoagulation due to previous CSC, history of anti-vascular endothelial growth factor injection treatment in either eye within 3 months, history of other retinal or choroidal diseases other than CSC, reduced OCTA image quality due to media opacity such as cataract (Topcon image quality index <50), and significant myopia (spherical equivalent <−8 D or axial length ≥26 mm). Moreover, cases of choroidal neovascularization (CNV) diagnosed based on OCTA findings and patients with a history of taking eplerenone were excluded.

Pre-PDT (baseline), subjects were also examined for chief complaints (subjective visual symptoms), symptom duration (period from the onset of subjective symptoms to PDT), and systemic diseases. The degree of subjective visual symptoms after PDT, in comparison with those at baseline, was investigated at every visit. Subjective visual symptoms mentioned by the subject at baseline were classified into four categories: decreased visual acuity, scotoma, metamorphopsia, and decreased visual acuity with scotoma. In general, CSC is known to complain of various and complex symptoms. Representatives include decreased visual acuity, blurred vision, relative central scotoma, metamorphosis, moderate dyschromatopsia, micropsia, and reduced contrast sensitivity [1]. The patients included in this study also complained about complex symptoms. After explaining the possible symptoms, the main symptom was chosen by themselves. Interestingly, all chosen the main symptoms were included in the four categories mentioned above. According to the 6 months post-PDT results, these were divided into two groups, “complete recovery” if the symptoms subsided completely and “incomplete recovery” if the symptoms were relieved incompletely or did not change.

Several OCT and OCTA variables at before and 1, 3, and 6 months after PDT were measured by two examiners (G.W.L. and K.J.C.) who were blinded to all medical information.

### Measurement of the image of the optical coherence tomography

An image of a raster scan with a size of 20° × 20° was obtained using OCT in the enhanced depth imaging mode. The central foveal thickness (CFT) was taken as the average of the distance between the vitreoretinal interface and photoreceptor outer segment, which passed perpendicular to the fovea on the horizontal and vertical scans. SRF height was defined as the average value of the distance between the inner border of the RPE and outer border of the interdigitation zone, passing perpendicular to the fovea on a horizontal and vertical scan. If hyperreflective material was present, it was used as the boundary of the SRF space. Subfoveal choroidal thickness (SFCT) was set as the average value of the distance between the outer border of the RPE and the sclerochoroidal junction, passing perpendicular to the fovea on horizontal and vertical scans. Before PDT, the presence of RPE detachment, hypertrophic outer retinal changes, RPE undulation, and disruption of the ellipsoid zone were identified. The CFT, SFCT, presence of RPE undulation, and disruption of the ellipsoid zone were also identified at 1, 3, and 6 months after PDT.

### The process of analysis of optical coherence tomography angiography

**Fig 1:**
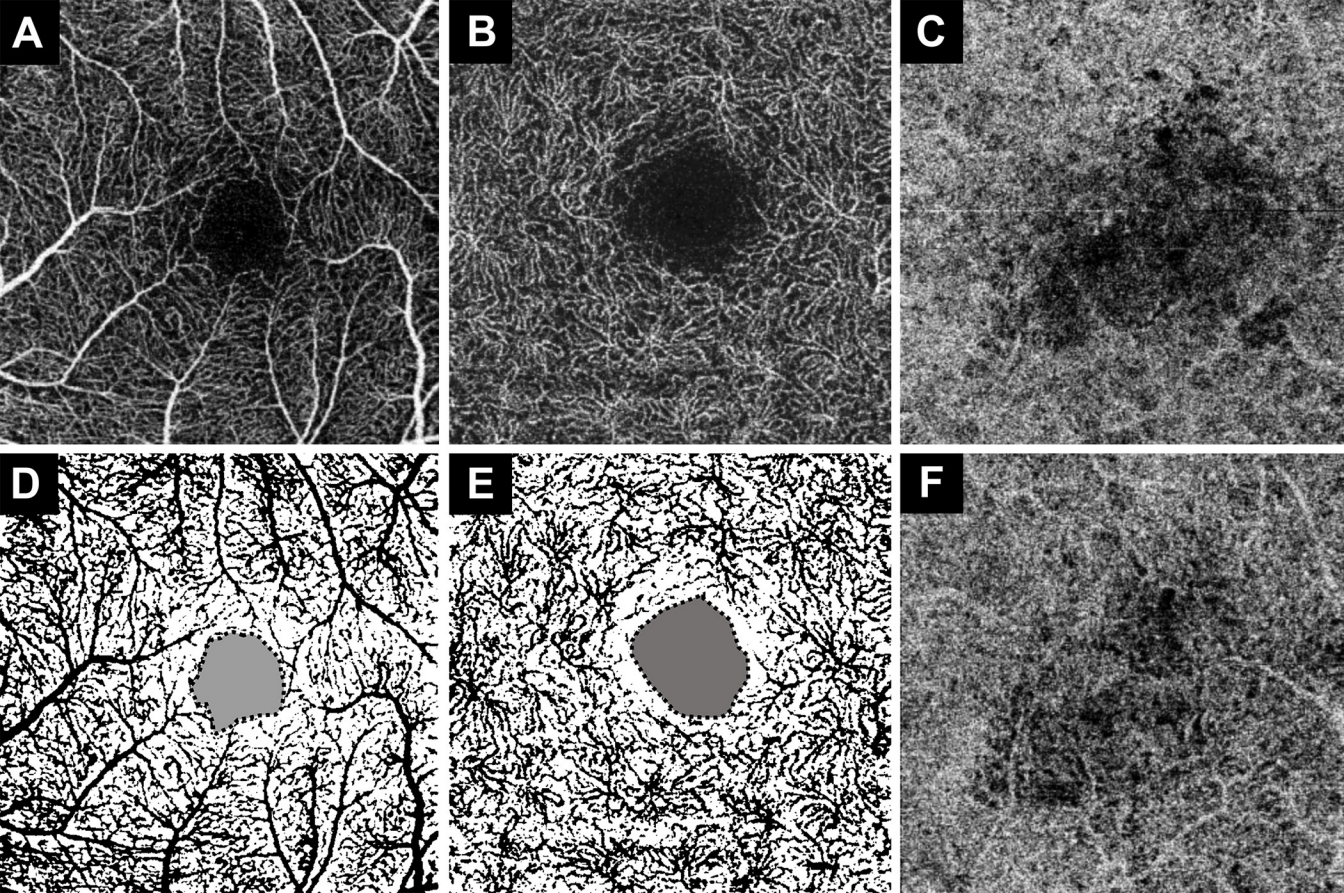
The analysis process of the en-face OCTA image. The 3 mm × 3 mm-sized en-face image of the SCP (A) and DCP (B) was converted to a binarized image (D and E) using ImageJ software. From the binarized image, the FAZ area (black dotted grayish area) was measured at the center, and the black colored vessel area was measured. Vessel density was obtained by dividing the vessel area by the area where the FAZ area was removed by 9 mm^2^. The signal void (F) of the choriocapillaris at 6 months post-PDT is smaller than the signal void (C) of the choriocapillaris after the subretinal fluid was completely absorbed 1 month post-PDT.

SS-OCTA images that automatically segmented the 3 × 3 mm^2^ macula area were identified using a viewing software. The en-face images of the superficial capillary plexus (SCP), deep capillary plexus (DCP), and choriocapillaris’ profiles were exported as quality-preserving JPEG files. Vessel density and the foveal avascular zone (FAZ) in the SCP and DCP were measured using an open-source software (ImageJ version 1.52a; National Institutes of Health, Bethesda, MD, USA; http://imagej.nih.gob/ij/). The FAZ was measured from the outline obtained using the polygon selection tool. The vessel density was calculated using methods similar to those reported previously (Fig 1D and 1E) [16,19]. Briefly, the area was converted to a binarized 8-bit image and measured to include the maximum number of vessels using threshold and binarization functions. The density was then obtained by dividing the area excluding the FAZ area by the total area of 9 mm^2^. In addition, two independent observers intuitively judged the signal void of choriocapillaris and divided them into two categories: “increasing or stationary” and “decreasing or resolved,” according to the comparison of the signal void between the time when the SRF had completely subsided (1 or 3 months post-PDT) and at 6 months post-PDT (Fig 1C and 1F). In cases with different results, a consensus was reached through discussion. The repeatability of the degree of change in the signal void was assessed using Cohen’s kappa coefficient. Cohen’s kappa coefficient was 0.788 (p < 0.001), which corresponds to substantial agreement.

### Photodynamic therapy procedure

All subjects included in this study underwent half-fluence PDT with verteporfin (Visudyne; Novartis, Basel, Switzerland). We used a standard dose of verteporfin (6 mg/m^2^), 689 nm laser, a light intensity of 600 mW/cm^2^, and a shortened irradiation time of approximately 40 s. The delivered radiation covered the area of choroidal hyperpermeability, which had engendered subfoveal SRF, in the mid or late phase of indocyanine green angiography.

### Statistical analysis

BCVA was converted to the logMAR scale, and manifest refraction was converted to a spherical equivalent for analyses. All continuous variables are reported as median (interquartile range). The Mann–Whitney *U* test was used to compare continuous variables between the two groups. To compare categorical variables between the two groups, cross tabulation analyses (chi-squared test) were used. For repeated measures, a generalized estimation equation was used to analyze the differences between groups and visits. In univariate analysis, when the p-value was less than 0.05, it was used as an explanatory variable for multivariate analysis, and multiple binary logistic regression was used for multivariate analysis. In addition, the cut-off value in the continuous variable showing a significant difference between the two groups was also checked through the receiver operating characteristic curve. And, spearman correlation analysis was performed to identify factors that correlated with BCVA or changes in BCVA.

## Results

**Fig 2:**
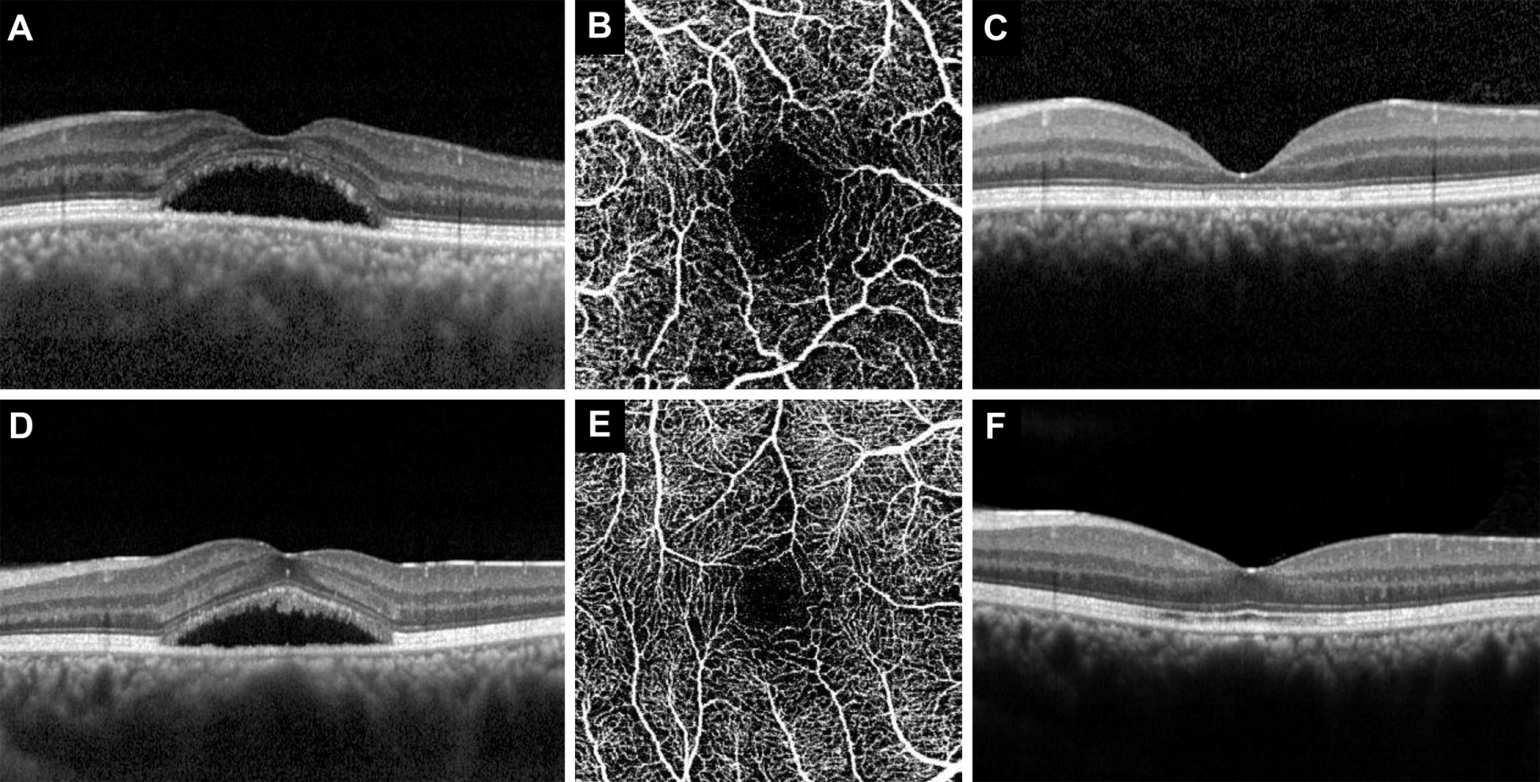
Horizontal section of OCT image and OCTA image of SCP of a 67-year-old man in the incomplete recovery group (A, B, C) and 63-year-old man in the complete recovery group (D, E, F). Compared with the 67-year-old man (incomplete recovery group), the 63-year-old man (complete recovery group) had better BCVA at baseline and 6 months after PDT (logMAR BCVA, 0.05 vs. 0.22 and 0.05 vs. 0.10, respectively), and shorter symptom duration (4 months vs. 7 months, respectively). Moreover, the central foveal thickness before (A, D) and after (C, F) PDT was thicker (182 μm vs. 145 μm and 201 μm vs. 135 μm, respectively), and the SCP FAZ area (B, E) before PDT was narrower (0.22 mm^2^ vs. 0.32 mm^2^, respectively).

Of the 69 eyes (69 CSC patients) examined retrospectively, 10 eyes met the exclusion criteria and 12 eyes were lost to follow-up. Among 47 eyes (47 CSC patients), five eyes that did not experience subsiding SRF up to 3 months after PDT and one eye with recurrence at 6 months were excluded from the study. Eventually, 42 eyes (42 patients with CSC) were analyzed (ratio of successful PDT = 89.4%). Demographics, OCT and OCTA measurements, and subjective visual symptoms before PDT (baseline) are presented in Table 1. The mean age of all subjects was 49.0(13) years, and included 32 men (76%) and 10 women (24%). At baseline, the mean logMAR BCVA was 0.10(0.16), and the mean CFT was 150(34)μm. The mean area of the SCP FAZ and DCP FAZ was 0.29(0.06)mm^2^ and 0.31(0.07)mm^2^, respectively, and mean vessel density of the SCP and DCP was 35.9(2.2)%, and 36.9(1.6)%, respectively. Regarding the frequency of the four subjective visual symptoms, 12 eyes (28.6%) had decreased visual acuity, 14 (33.3%) had scotoma, 14 (33.3%) had decreased visual acuity and scotoma, and 2 (4.8%) had metamorphopsia. When comparing the subjective visual symptoms at baseline and 6 months after PDT, patients in whom symptoms completely disappeared were included in the complete recovery group (20 eyes). Patients in whom symptoms did not disappear were included in the incomplete recovery group (22 eyes). The complete recovery group before PDT had greater CFT than that of the incomplete recovery group (p < 0.01). In addition, in the complete recovery group, SCP FAZ and DCP FAZ were smaller (all p < 0.01). Fig 2 shows representative cases for the two groups.

**Table 1:** Comparison of baseline demographics and clinical characteristics between the two groups.

Variable	Total (n = 42)	Complete recovery group (n = 20)	Incomplete recovery group (n = 22)	p-value
Age, years	49.0 (13)	47.0 (16)	50.5 (8)	0.371
Sex, M:F	32:10	17:3	15:7	0.284
Symptom duration, months	5 (3)	3.5(3)	5(3)	0.058
Systemic disease				
Diabetes mellitus, n (%)	4(9.5)	3(15.0)	1(4.5)	0.333
Hypertension, n (%)	2(10.0)	0(0)	2(4.8)	0.221
BCVA, logMAR	0.10 (0.16)	0.05(0.14)	0.10(0.11)	0.139
Spherical equivalent	0 (6)	0 (1)	0 (1.5)	0.654
OCT measurements				
CFT, μm	150 (34)	159 (46)	131(48)	<0.001
SRF height, μm	123 (72)	113 (91)	147 (31)	0.202
SFCT, μm	401 (81.5)	402.5 (60)	385.5(88)	0.155
Presence of PED, n (%)	9(21.4)	5(25.0)	4(18.2)	0.714
RPE undulation, n (%)	18(42.9)	8(40.0)	10(45.5)	0.764
Hypertrophic outer retinal change, n (%)	27(64.3)	4(80.0)	11(50.0)	0.082
OCTA measurements				
SCP FAZ, mm^2^	0.29 (0.06)	0.25(0.07)	0.31(0.04)	<0.001
SCP VD, %	35.9(2.2)	35.7(2.3)	35.9(2.1)	0.860
DCP FAZ, mm^2^	0.31(0.07)	0.28(0.09)	0.34(0.06)	0.005
DCP VD, %	36.9(1.6)	36.5(1.9)	37.1(1.7)	0.623
Visual symptoms at diagnosis				
Decreased visual acuity, n (%)	12 (28.6)	9 (40.0)	4 (18.2)	0.175
Scotoma, n (%)	14 (33.3)	7 (35.0)	7 (31.8)	1.000
Decreased VA & scotoma, n (%)	14 (33.3)	5 (25.0)	9 (40.9)	0.338
Metamorphopsia, n (%)	2 (4.8)	0 (0)	2 (9.1)	0.489

p-values were obtained by mann–whitney *U* test and pearson’s chi-squared test.

Symptom duration refers to the period from occurrence of subjective symptom to PDT.

All continuous variables were presented by median(interquartile range).

Abbreviations: CSC, central serous chorioretinopathy; BCVA, best corrected visual acuity; logMAR, logarithm of the minimum angle resolution; OCT, optical coherence tomography; OCTA, optical coherence tomography angiography; CFT, central foveal thickness; SRF, subretinal fluid; SFCT, subfoveal choroidal thickness; PED, pigment epithelial detachment; RPE, retinal pigment epithelium; SCP, superficial capillary plexus; DCP, deep capillary plexus; FAZ, foveal avascular zone; VD, vessel density.

A generalized estimation equation has been used to investigate the change in repeated measures according to visits and groups (Table 2). BCVA, CFT, SFCT, SCP FAZ, and DCP FAZ were analyzed. Further, the differences according to visits and groups, separately and simultaneously, and after adjusting for the age, sex, and symptom duration were comprehensively analyzed. Although the groups did not differ in BCVA or choroidal thickness, the differences with visits were significant (all *p* < 0.05). For CFT, SCP FAZ, and DCP FAZ, differences among visits were insignificant but those among groups were significant (all p < 0.01).

**Table 2:** Comparison of sequential characteristics by groups and by the visit.

Variable	Visit	Complete recovery group	Incomplete recovery group	p-value(Crude)			p-value(Adjusted) †		
				V	G	V*G	V	G	V*G
BCVA, logMAR	Baseline	0.05(0–0.13)	0.13(0.05–0.22)	< .001	0.139	0.433	< .001	0.11	0.427
	1 month	0(0–0.07)	0.05(0–0.1)						
	3 months	0(0–0)	0(0–0.1)						
	6 months	0(0–0.05)	0(0–0.05)						
CFT, μm	Baseline	159(152.5–174.5)	130.5(102–150)	0.092	< .001	0.063	0.087	< .001	0.067
	1 month	158.5(147.5–175)	142.5(131–146)						
	3 months	166.5(150–178.5)	142(121–158)						
	6 months	164.5(154–180)	148(112–162)						
SFCT, μm	Baseline	391(389–438)	385.5(341–422)	0.048	0.253	0.059	0.038	0.157	0.095
	1 month	377(339.5–425)	397(333–433)						
	3 months	379(318.5–460.5)	346(321–376)						
	6 months	368(339.5–425)	366.5(330–415)						
SCP FAZ, mm^2^	Baseline	0.25(0.23–0.30)	0.31(0.29–0.33)	0.389	< .001	0.343	0.397	< .001	0.42
	1 month	0.25(0.24–0.31)	0.32(0.30–0.33)						
	3 months	0.25(0.25–0.32)	0.31(0.30–0.33)						
	6 months	0.26(0.24–0.30)	0.31(0.29–0.33)						
DCP FAZ, mm^2^	Baseline	0.28(0.27–0.36)	0.34(0.31–0.36)	0.083	< .001	0.078	0.076	< .001	0.084
	1 month	0.29(0.28–0.32)	0.33(0.31–0.38)						
	3 months	0.30(0.28–0.33)	0.33(0.32–0.38)						
	6 months	0.30(0.28–0.33)	0.33(0.32–0.38)						

Abbreviations: V, visit; G, group.

All values were presented by median (interquartile range).

p-values were obtained by generalized estimation equation (GEE).

†: Adjusted results were obtained with covariate variable as age(years), sex and symptom duration(months).

**Fig 3:**
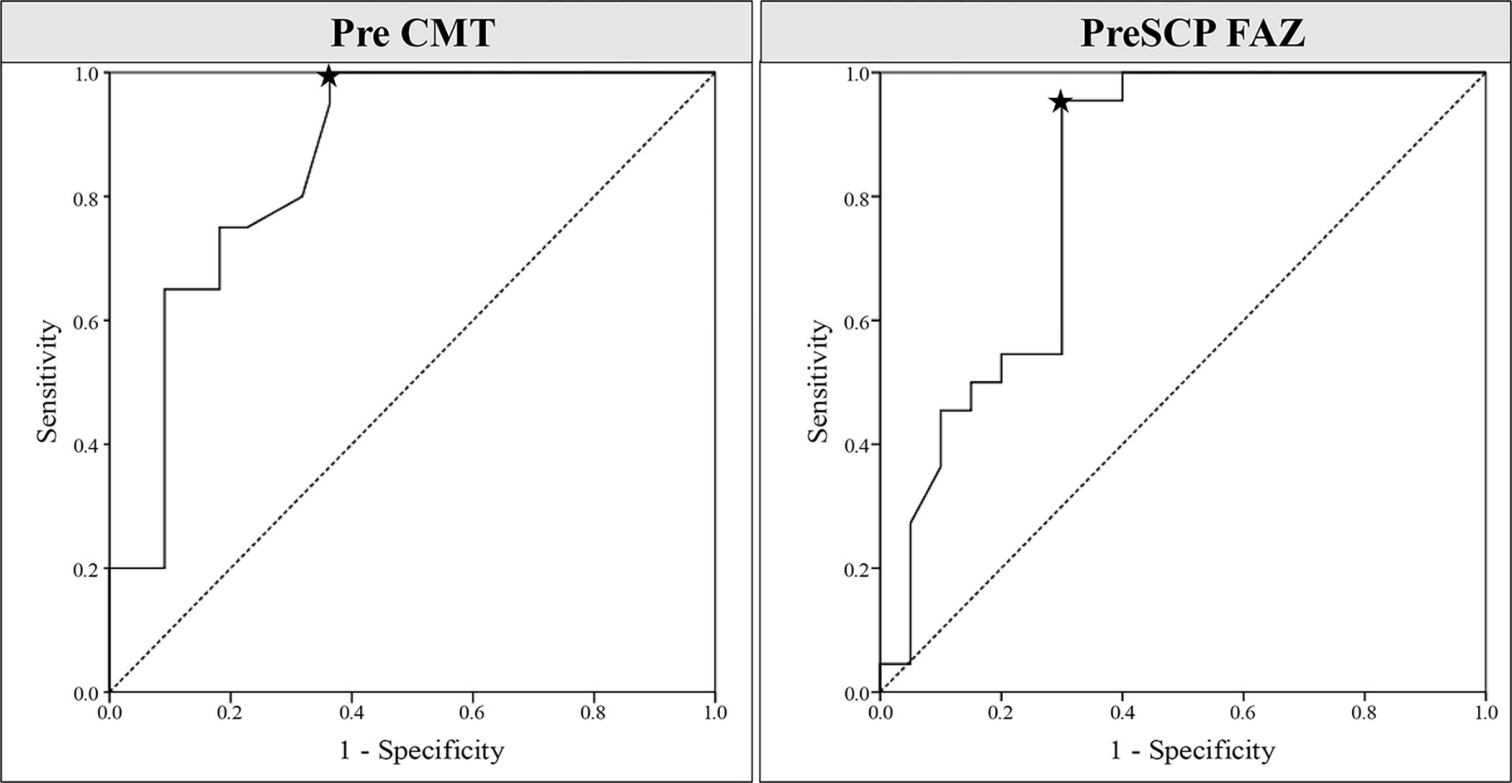
Receiver operating characteristic curves of baseline CFT and SCP-FAZ. The area under the curve was 0.858 for CFT and 0.816 for SCP FAZ. The cutoff value (asterisk) using the Youden index was 139.5 μm for CFT and 0.265 mm^2^ for SCP FAZ. CFT, central foveal thickness; SCP, superficial capillary plexus; FAZ, foveal avascular zone.

When the signal void of the choriocapillaris at 6 months after PDT was compared with the signal void immediately after the resolution of SRF, the ratio of subjects whose signal void was reduced or disappeared was greater in the complete recovery group (p = 0.012).Multiple binary logistic regression analyses were performed to identify the independent factors associated with the recovery of subjective visual symptoms. Variables with a p-value < 0.05 in the univariate analysis were used as explanatory variables in the multivariate analysis. The explanatory variables satisfying the condition were CFT, SCP FAZ, and DCP FAZ (all p < 0.01). Because of the high correlation among explanatory variables that were significant in the univariate analysis, forward (condition) was used as the variable selection method to resolve multicollinearity. In multivariate analysis, only 1 variable(CFT) was selected, and when CFT increased by 1 unit, the odds ratio of incomplete recovery was 0.931 times higher than that of complete recovery (95% confidence interval: 0.890 to 0.975, p = 0.002). Among the factors differing significantly between the two groups, the cutoff CFT and SCP FAZ (using the Youden index) were obtained from the receiver operating characteristic curve (Fig 3). Recovery of subjective symptoms was more likely to be incomplete when CFT was < 139.5 μm or SCP FAZ was > 0.265 mm^2^.

**Fig 4:**
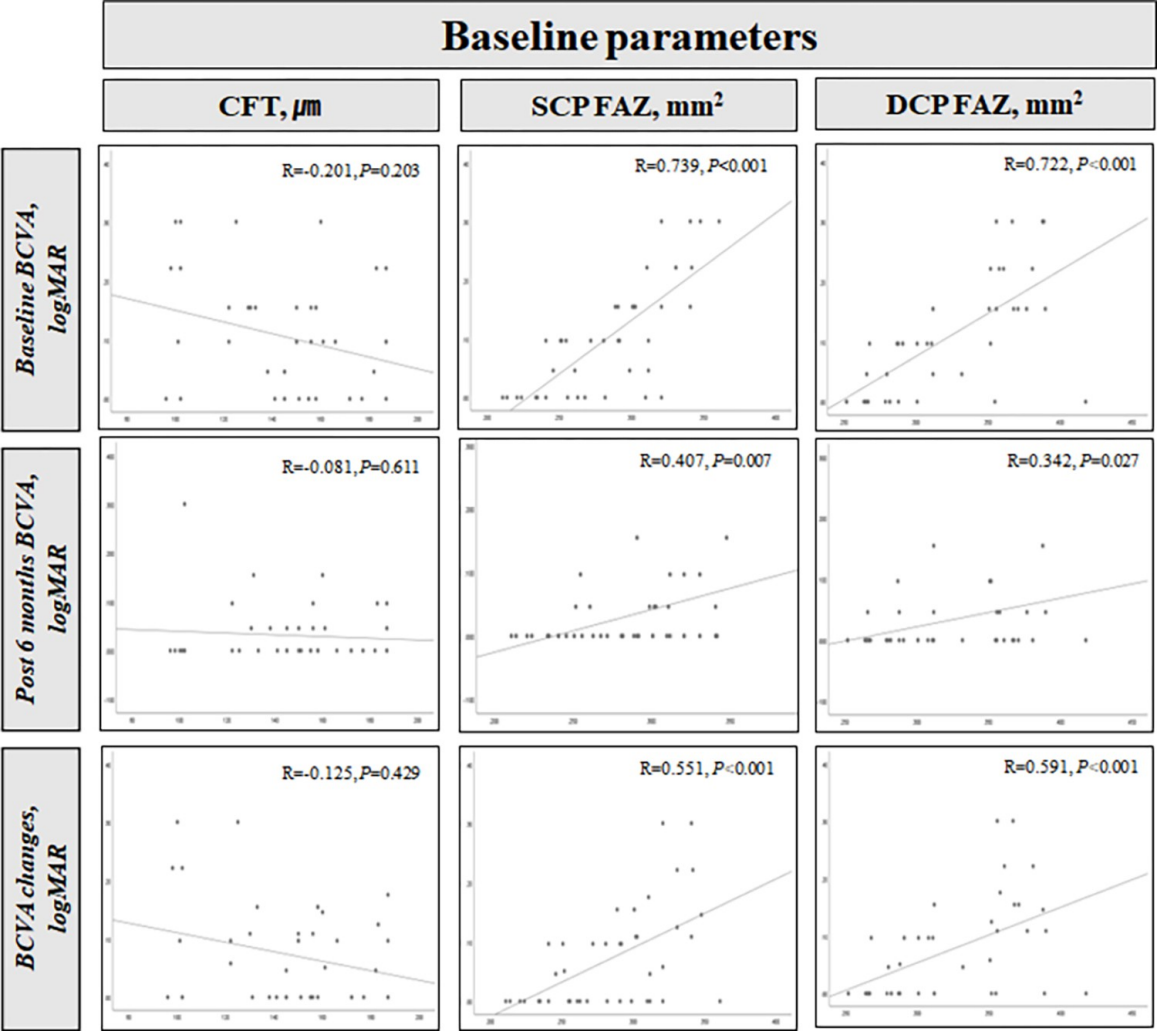
Graphs showing the correlation between variables before PDT (CFT, SCP FAZ, and DCP FAZ) and baseline BCVA, 6 months post-PDT BCVA, and BCVA changes over 6 months. BCVA-related variables show a significant correlation with both SCP FAZ and DCP FAZ (all p < 0.01). CFT, central foveal thickness; SCP, superficial capillary plexus; DCP, deep capillary plexus; FAZ, foveal avascular zone; R, Spearman’s rho coefficient; BCVA, best corrected visual acuity; logMAR, logarithm of the minimum angle of resolution; PDT, photodynamic therapy.

Among the measures of CFT, SCP FAZ, and DCP FAZ at baseline, the factors related to BCVA were investigated (Fig 4). Baseline BCVA, 6 months post-PDT BCVA, and the amount of BCVA changes over 6 months showed a significant relationship with SCP FAZ and DCP FAZ at baseline. In other words, as the FAZ area was wider, baseline BCVA and 6 months post-PDT BCVA were worse; however, the magnitude of BCVA improvement was greater.

## Discussion

PDT is a treatment involving irradiation of lesions visible on angiography with a laser after verteporfin injection, known to be effective in chronic CSC. It is presumed that free radicals generated under the damaged RPE site damage the vascular endothelium, resulting in hypoperfusion and remodeling of the choriocapillaris [20]. In this regard, side effects, such as decreased visual acuity and visual field impairment, have been reported after standard full-setting PDT. Recently, to lower the risk of side effects, reduced dose and reduced time PDT have been mainly used and reported to be effective treatments [21–23].

In several previous studies, metamorphopsia, and not visual acuity, was reported to be a factor significantly associated with vision-related quality of life in diseases involving the macula (e.g., epiretinal membrane, macular hole, macular-off retinal detachment) [24–26]. In other words, there are visual symptoms that cause discomfort to patients’ and interfere with their daily life much more than decreased visual acuity. We focused on these points to investigate the changes in subjective visual symptoms. We divided the patients into two groups to determine the factors that influenced the change in subjective visual symptoms. Those with complete disappearance of subjective visual symptoms six months after successful PDT were classified into the complete response group. In this study, this included 20 (47.6%) out of total 42 eyes. After PDT, the all subjects showed a significant increase in BCVA, but there was no significant difference in BCVA between the two groups. This means that visual acuity does not sufficiently represent changes in visual symptoms, as reported in previous studies.

OCT and OCTA have also been used to examine changes that occur after PDT in CSC, in previous studies. Demircan et al. reported that hypoperfusion of the choriocapillaris on OCTA was clearly observed on the 3rd day after PDT, but returned to normal after 1 month [14]. Demirel et al. reported that choriocapillaris flow increased and total choroidal area decreased at 1 month after PDT, but FAZ area and vessel density did not show any significant difference from those at baseline [17]. Similar to this study, Rochepeau et al. reported that SRF could be a false-positive source, so the degree of signal void was compared with the opposite eye after the SRF subsided, and that the signal void was significantly larger than the opposite eye [27]. Furthermore, Rabiolo et al. also reported that choroidal thickness decreased at 3 months after PDT, but did not show any significant change in the FAZ area [16]. Similar to previous studies, after PDT, the choroid thickness decreased significantly and the FAZ area did not show any significant difference. When compared with the choriocapillaris at the time when SRF disappeared and the choriocapillaris at 6 months after PDT, the signal void was reduced in 31 out of 42 eyes (73.8%).

Gawecki et al reported that retinal thinning, visual impairment, and choroidal flow defects were observed in resolved chronic CSC [28]. Similarly, in our study, the wider the FAZ area, and the lower the CFT, the higher was the likelihood of having limited recovery from subjective visual symptoms. The larger the FAZ area before PDT, the lower was the BCVA before PDT and 6 months after PDT. In other words, as CSC becomes more chronic, subjective visual symptoms may remain and BCVA may be lower [12]. Haga et al. reported that younger age and better baseline BCVA are more likely to lead to successful treatment without recurrence [29]. In addition, Fujita et al. reported that BCVA was significantly increased after PDT, but PDT was less effective when BCVA was low before PDT [30]. Similar to previous studies, early PDT may be effective in the recovery of BCVA and subjective visual symptoms.

In addition to PDT, which is a representative treatment option for CSC, eplerenone may be an effective treatment [31]. A study on overactivation of the mineralocorticoid receptor inducing a choroidal pathology close to that of the pachychoroid showed that the oral drug eplerenone (mineralocorticoid receptor antagonist) decreases the choroidal blood flow and volume. The choroidal thickness varies with the axial length and examination time, while the choroidal vascular index is constant [31–34]. Toto et al. reported that oral eplerenone effectively lowered the choroidal vascular index in chronic CSC [31]. In this study, the subfoveal choroidal thickness and degree of CC signal void change in the choroid were used. The subfoveal choroidal thickness showed a tendency to decrease with PDT treatment, but the difference between the two groups was insignificant. However, a limitation of the study was the lack of consideration of the measurement time and axial length. Further, the recovery of the signal void 6 months after PDT was better in the complete response group, under the limitation of a lack of measurement results. In the future, an analysis should be performed using the choroidal vascular index in addition to the retinal vessel density.

Sulzbacher et al. reported that the visual prognosis of neovascular CSCs with CNV detection on OCTA was poor [35]. In this study, visual symptoms and acuity were also checked, but patients with confirmed CNV were excluded from the study. In a previous long-term study, the development of CNV was higher in the incomplete response group, consistent with our study. Novel conclusions may be drawn if we observe the progression over a longer period.

There are two clinically important facts suggested by these results. First, in patients with CSC, those with low CFT or large FAZ of SCP, DCP may not fully recover from their symptoms after PDT and may have sequelae, patients should be informed of this possibility before treatment. Second, and more importantly, although waiting for natural recovery in CSC patients may be a legitimate form of treatment, it would be better not to postpone PDT until CFT decreases or FAZ increases. Although the change in visual acuity is a major consideration in determining the initiation of PDT treatment, this study suggests that visual complaints may remain even with good visual acuity. In other words, CFT and FAZ should be major considerations in treatment decisions. Even if the visual acuity is good, relatively early intervention before the changes in CFT and FAZ become more profound appears preferable; however, further research is needed in this regard.

The limitations of this study are that the sample size was small and the follow-up period was short. Another potential issue is that the groups were defined using subjective symptoms directly reported by the subjects. The severity of symptoms was not considered as it was difficult to measure because of the retrospective nature of the study. However, the analysis of chief complaints would have some degree of reliability. A prospective study on symptom severity should be conducted in the future. Finally, of a total of 69 eyes, 42 (60.8%) were included in the analysis. Since 12 of the 27 excluded eyes had been lost to follow-up, selection bias may have occurred. This was a limitation of the retrospective study design. Nevertheless, this is the first study to investigate the differences in clinical features, including OCT and OCTA, and their relationship with changes in subjective visual symptoms after PDT.

In conclusion, before PDT, the thicker the CFT and the smaller the FAZ area, the easier it is to recover from subjective visual symptoms. Before PDT, the wider the FAZ area, the lower the BCVA before and 6 months after PDT. These facts can be meaningful in explaining the degree of recovery from subjective visual symptoms after PDT for patients before PDT.

## Supporting information

S1 Dataset (XLSX)
